# Surgical Blowhole Incision for Acute Subcutaneous Emphysema: A Novel Approach With Negative-Pressure Wound Therapy

**DOI:** 10.7759/cureus.93733

**Published:** 2025-10-02

**Authors:** Scott W Brown, TristaDawn J Ison, Morgan J Holko, Nicholas Bahl

**Affiliations:** 1 Emergency Medicine, Pacific Northwest University of Health Sciences, Yakima, USA; 2 Surgery, Regional Medical Center Bayonet Point, Hudson, USA

**Keywords:** blowhole incision, hemopneumothorax, incisional negative pressure wound therapy, periorbital edema, pulmonary critical care, subcutaneous emphysema management, traumatic pneumomediastinum

## Abstract

This case report describes a patient who sustained significant subcutaneous emphysema of the chest, neck, and face, along with hemopneumothorax and multiple left-sided rib fractures with an associated flail segment following an all-terrain vehicle accident. The emphysema was treated using negative pressure wound therapy (NPWT) applied through bilateral infraclavicular “blowhole” incisions. This report examines the use of NPWT as a treatment modality for subcutaneous emphysema, assessing its efficacy in reducing the need for invasive interventions and promoting recovery. The patient’s clinical presentation, NPWT application technique, and treatment progression were documented. Additionally, a literature review was conducted to contextualize NPWT’s role in managing subcutaneous emphysema. The patient exhibited a marked reduction in subcutaneous emphysema within 24 hours of NPWT initiation, with continued improvement confirmed through physical examinations and imaging. By day 13, complete resolution was achieved, and NPWT was discontinued without complications. This case highlights NPWT as a promising, minimally invasive treatment option for subcutaneous emphysema, demonstrating rapid resolution and favorable outcomes. Further studies may help define its broader clinical applications.

## Introduction

Subcutaneous emphysema (SE) is a relatively rare condition characterized by air infiltrating beneath the subcutaneous layer of the skin. While often benign, SE can progress into deeper tissue layers, and in some cases, air within other body cavities can expand outwards, causing SE, such as in pneumomediastinum, pneumoperitoneum, pneumoretroperitoneum, or pneumothorax. In the setting of lung injury, air can travel along pressure gradients between the alveoli and perivascular interstitium to the neck, head, or abdomen. Air naturally accumulates in subcutaneous tissue with the least amount of tension; however, unchecked air accumulation can infiltrate into adjacent plans, potentially leading to severe complications such as cardiovascular and respiratory collapse. Such extreme cases of subcutaneous emphysema are dependent on the source or inciting event [1].

Common causes of subcutaneous emphysema include blunt trauma, such as rib fractures or crush injuries; severe infections that place extreme stress on the respiratory system; iatrogenic disease; and, lastly, idiopathic origins [1,2]. SE can be classified, though not typically clinically utilized, into five categories based on anatomical location: (1) the base of the neck, (2) the entire neck, (3) involvement of the pectoralis major, (4) the chest wall and the entire neck, and (5) extensive SE, extending from the scalp to the scrotum. The most common cause of grade 5 is pneumothorax in the setting of chronic obstructive pulmonary disease (COPD) or recent surgery [2]. Iatrogenic factors, particularly laparoscopic procedures utilizing carbon dioxide insufflation, represent an additional risk factor for developing SE. Treatment options for persistent pneumothorax may include video-assisted thoracic surgery (VATS); however, this approach can be counterintuitive, as the treatment involves utilizing carbon dioxide insufflation into the thoracic cavity [3].

Minor cases of SE are often treated conservatively, relying on watchful waiting; however, widespread SE requires more invasive therapy to prevent further complications and preserve cardiac and respiratory function [4]. Treatment should focus on managing the underlying cause, with the first step being an appropriately placed intercostal drain. For the underlying pneumothorax, negative suction, wider bore drains, and surgical management should be employed. Specific SE treatments include infraclavicular "blowhole" incisions, subcutaneous angio-catheters, or tunneled drains [5,6]. Negative suction on an existing intercostal drain has also been proven effective without the need of additional modalities [3]. The most effective treatment modality varies, with infraclavicular incisions, drain insertion, and increased suction on in-situ drains all shown to provide rapid relief. The following case study highlights a scenario in which conservative management failed to adequately address a hemopneumothorax and SE, necessitating intervention via blowhole incision and NPWT to expedite recovery.

## Case presentation

A 62-year-old male presented to the emergency department with a chief complaint of severe left-sided chest pain following an all-terrain vehicle (ATV) accident. The patient reported that he had been riding an ATV at home when he turned sharply, causing the vehicle to tip over and land on his left side. He described the pain as severe (10/10), non-radiating, and exacerbated by breathing. He denied any pain or tenderness in his extremities or abdomen and reported no loss of consciousness, vomiting, headache, dizziness, tingling, numbness, or uncontrolled bleeding. However, he acknowledged being under the influence of alcohol at the time of the accident and was not wearing a helmet. He denied any history of syncope or prior head injuries.

The patient was discovered trapped under the ATV by an elderly neighbor, who promptly alerted emergency services. Upon arrival at the emergency department, the patient appeared disheveled, agitated, and in significant distress. Physical examination revealed multiple abrasions and ecchymoses along the bilateral knees, left flank, left chest, and upper extremities. Notably, there was an 8-cm oblique laceration on the left supraorbital ridge, along with marked bilateral periorbital edema. Respiratory examination showed labored breathing with pronounced subcutaneous emphysema.

The patient’s past medical history was significant for essential hypertension, controlled with lisinopril. He had no significant surgical history. His social history included regular alcohol and marijuana use. A review of systems revealed chest pain, bilateral periorbital edema, and a nonproductive cough.

**Figure 1:**
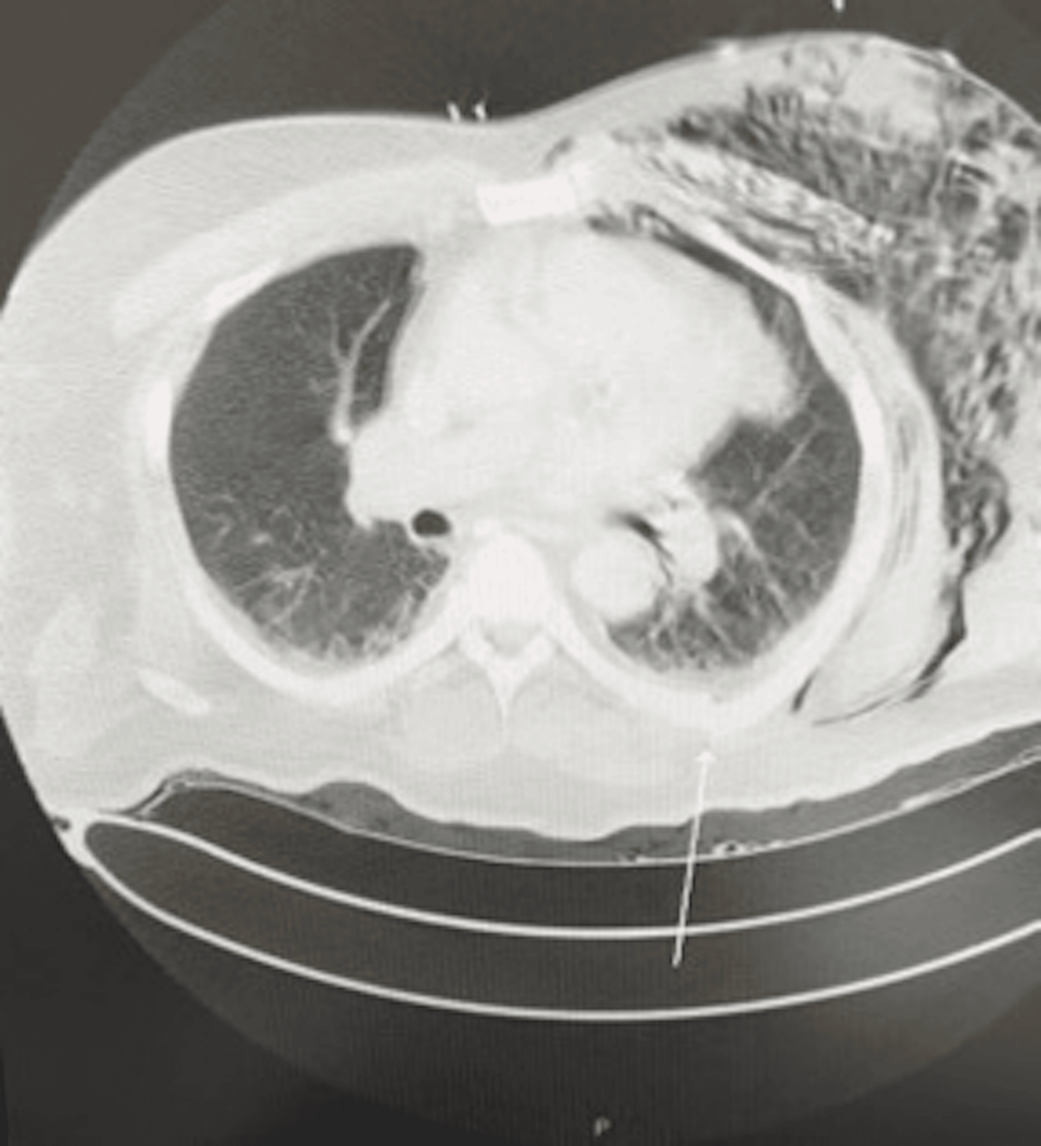
Computed tomography chest imaging demonstrating large volume subcutaneous emphysema, trace left hemopneumothorax, and posterior medial fracture of the sixth rib (white arrow).

Initial chest X-ray in the ED revealed multiple left rib fractures with subcutaneous emphysema with no pneumothorax. Once stabilized, a computed tomography (CT) scan of the chest, abdomen, and pelvis (Figure 1) revealed acute fractures involving the left anterolateral second to seventh ribs and the posterior medial left third to sixth ribs. Additionally, a small left-sided pneumothorax was identified, along with extensive soft tissue emphysema extending along the left chest wall and axilla. General surgery and interventional radiology were consulted for chest tube placement, but believed conservative management was indicated.

**Figure 2:**
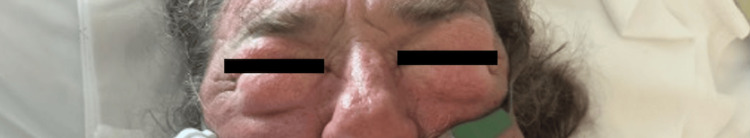
This image illustrates the patient's appearance upon admission to the ICU, with pronounced bilateral periorbital edema and compromised respiratory status requiring high-flow nasal cannula. The forehead demonstrates a repaired 8-cm oblique laceration along the left supraorbital ridge, closed with interrupted 4-0 nylon sutures.

In the emergency department, the patient's forehead laceration was repaired, and deep vein thrombosis (DVT) prophylaxis was initiated. Given the severity of his injuries and his compromised respiratory status (Figure 2), the patient was admitted to the intensive care unit (ICU) for close monitoring and further management.

The following morning, repeat chest X-ray demonstrated worsening subcutaneous emphysema with no evidence of pneumothorax. A repeat chest CT revealed bilateral, worsening subcutaneous emphysema with no evidence of pneumothorax. Considering the progression of his condition, additional interventions were required to manage his respiratory distress.

**Figure 3:**
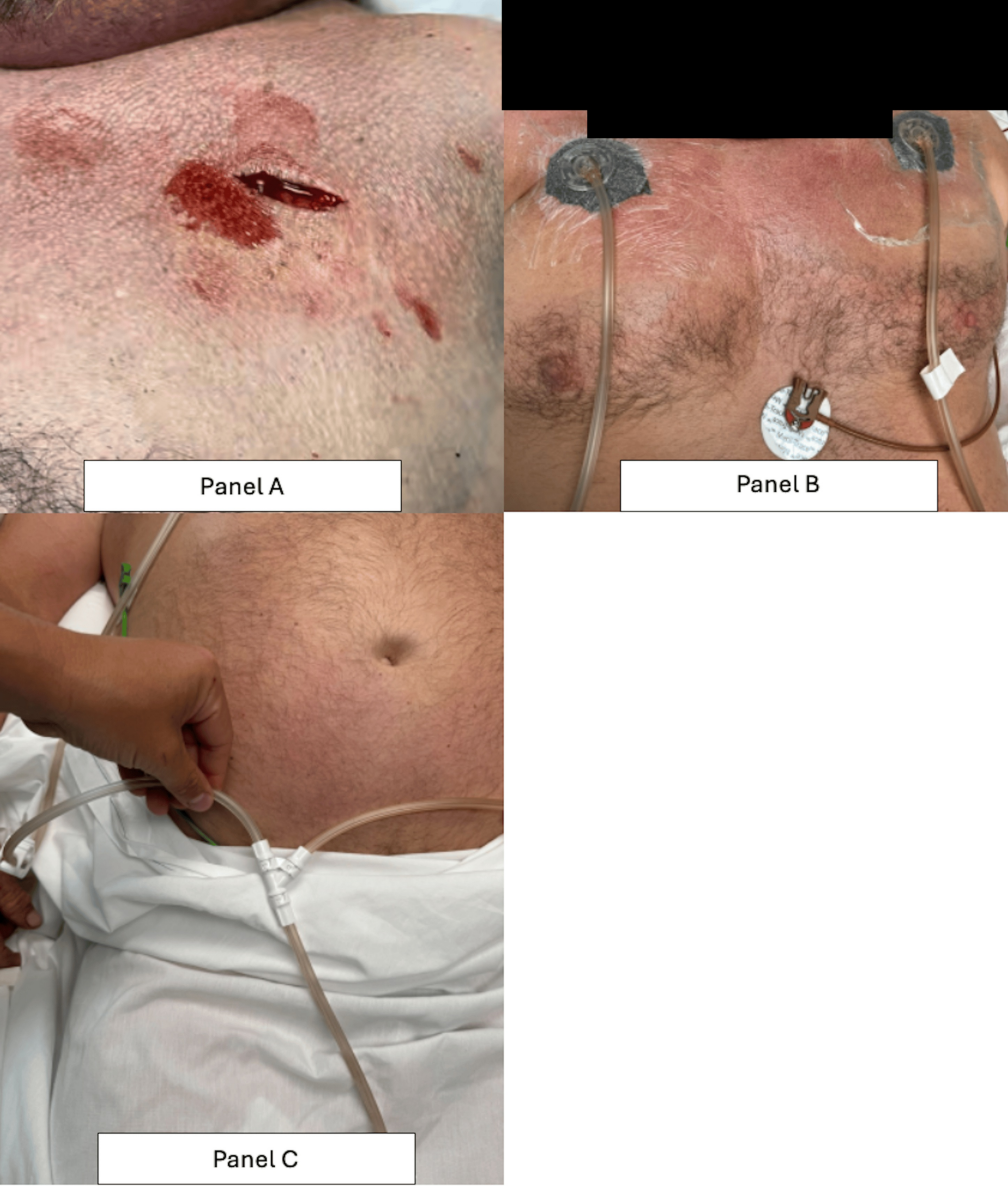
Blowhole incisions with negative-pressure wound therapy applied bilaterally and connected with a Y-connector for equal pressure distribution. (A) Left infraclavicular incision located 4-5 cm inferior to the clavicle, with a corresponding incision on the right chest wall, facilitating air evacuation from the subcutaneous space. (B) Postoperative day 0 immediately following the procedure showing negative pressure wound vacuums (NPWTs) in place with circular foam dressings, approximately 3 cm in diameter, secured with a film seal. (C) NPWT tubing joined via a Y-connector to ensure equal pressure distribution across bilateral wound sites.

The intervention performed was a bilateral infraclavicular subcutaneous incision, coined “blowhole incision.” Prior to incision, 10 cc of 1% lidocaine with epinephrine was utilized for local field anesthesia. Using a #10 scalpel blade, a 4- to 5-cm incision was made below both clavicles. The subcutaneous tissue was carefully dissected with a #15 scalpel, and a pocket was created down to the pectoralis fascia using finger dissection. Negative pressure wound vacuum (NPWT) was applied to facilitate the evacuation of air and fluids from the subcutaneous space (Figures 3A-3C).

**Figure 4:**
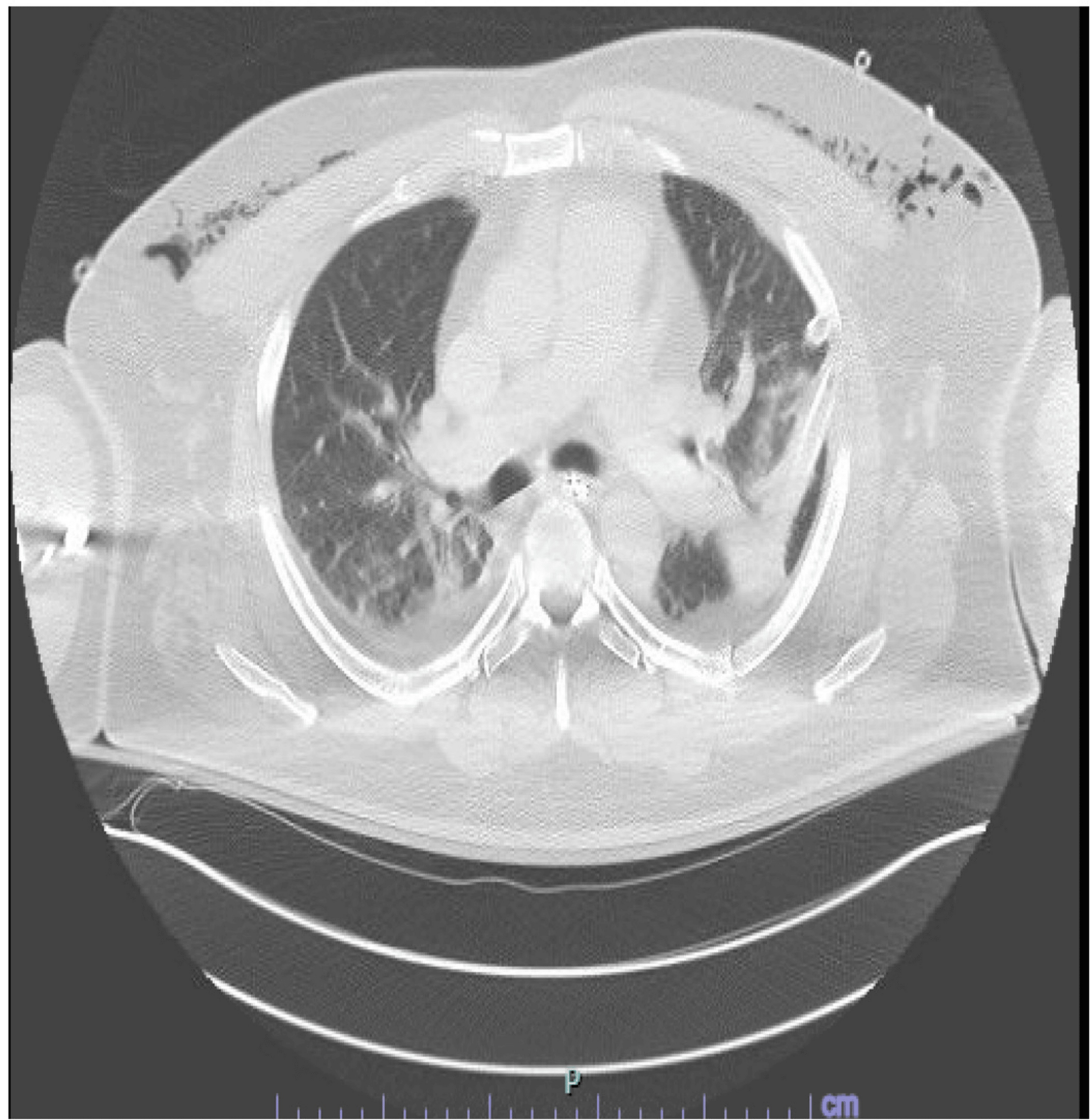
A repeat chest CT performed on postoperative day 1 following NPWT placement, showing improvement in subcutaneous emphysema and moderate pneumomediastinum. Rib fractures and small amounts of right and left pleural fluid, as observed on prior CT imaging, remained unchanged. NPWT: negative pressure wound vacuum.

The following morning, a third chest X-ray revealed substantially decreased subcutaneous emphysema with no evidence of pneumothorax. Repeat CT imaging revealed a decrease in SE (Figure 4), and the patient's vital signs demonstrated improvement in respiratory status. CT also revealed a small left-sided pneumothorax, which prompted interventional radiology consultation, who reported no indication for chest tube placement. This intervention remained in place for 13 days with complete resolution of the SE, with removal and placement of wet to dry dressings followed by adhesive wound closure strip.

**Figure 5:**
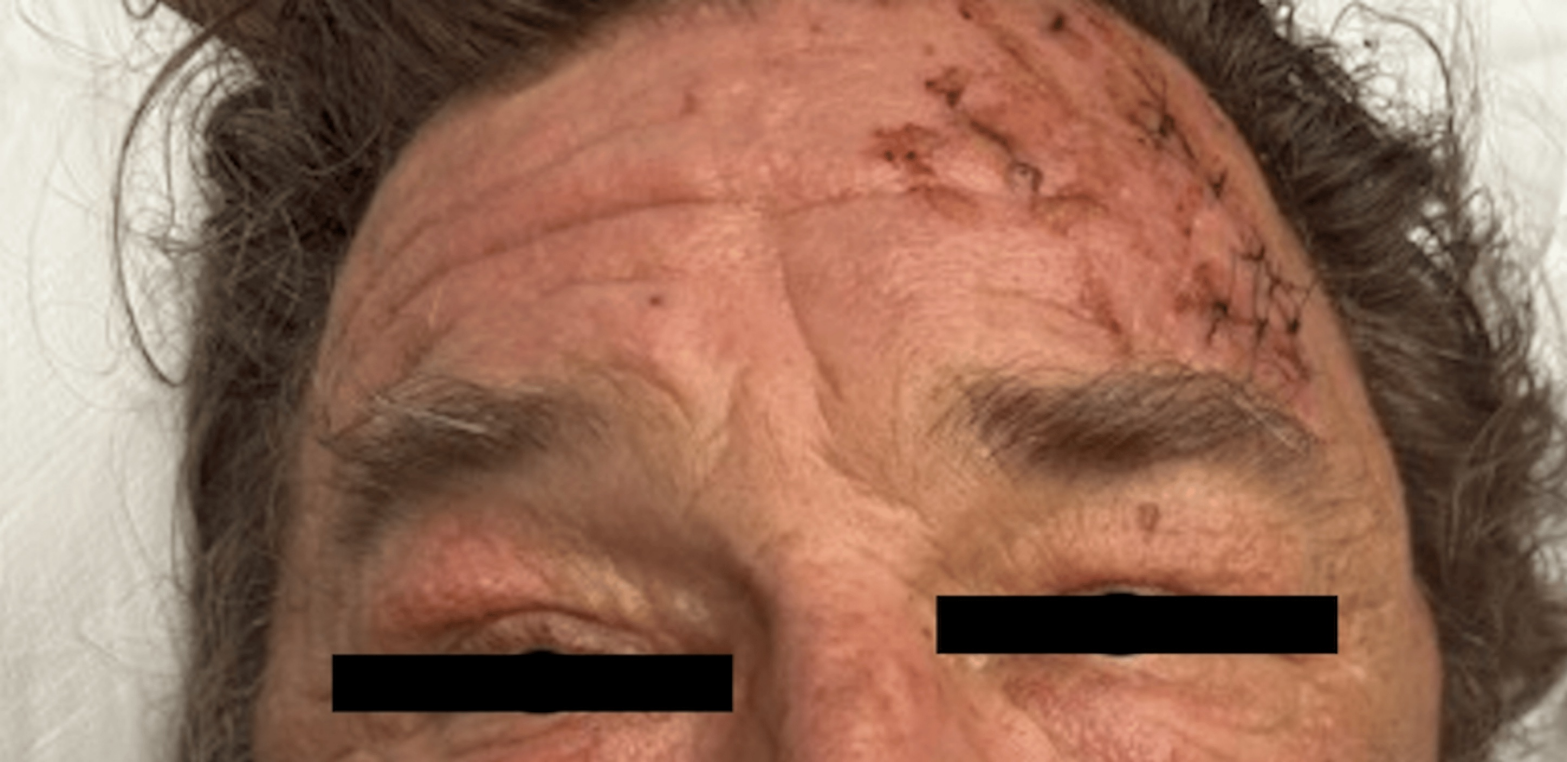
Postoperative day 1 with significant reduction in periorbital edema and subcutaneous emphysema with NPWT in place. NPWT: negative pressure wound vacuum.

Although the patient initially demonstrated significant improvement in subcutaneous emphysema following blowhole incisions with NPWT (Figure 5), the patient developed respiratory distress postoperative day 4 due to multiple factors, including agitated delirium exacerbated by alcohol and substance use, hemopneumothorax, and a flail chest segment. These complications impaired pulmonary hygiene, increased the risk of aspiration, and led to escalating fraction of inspired oxygen (FIO₂) requirements, ultimately necessitating intubation. While rib plating for the flail segment was considered, the patient’s respiratory status remained the primary concern.

A chest X-ray was ordered and showed signs of a new left-sided pleural effusion. In addition to this, a chest computed tomographic angiography (CTA) was ordered to assess for other causes of patient's respiratory status, including pulmonary embolism. It revealed a new left-sided pleural effusion concerning for hemothorax. Given the patient's deteriorating mental status, respiratory distress, and new-onset fever, placement of a right femoral intravascular temperature management system and left chest tube was deemed necessary for pleural fluid drainage and culture.

**Figure 6:**
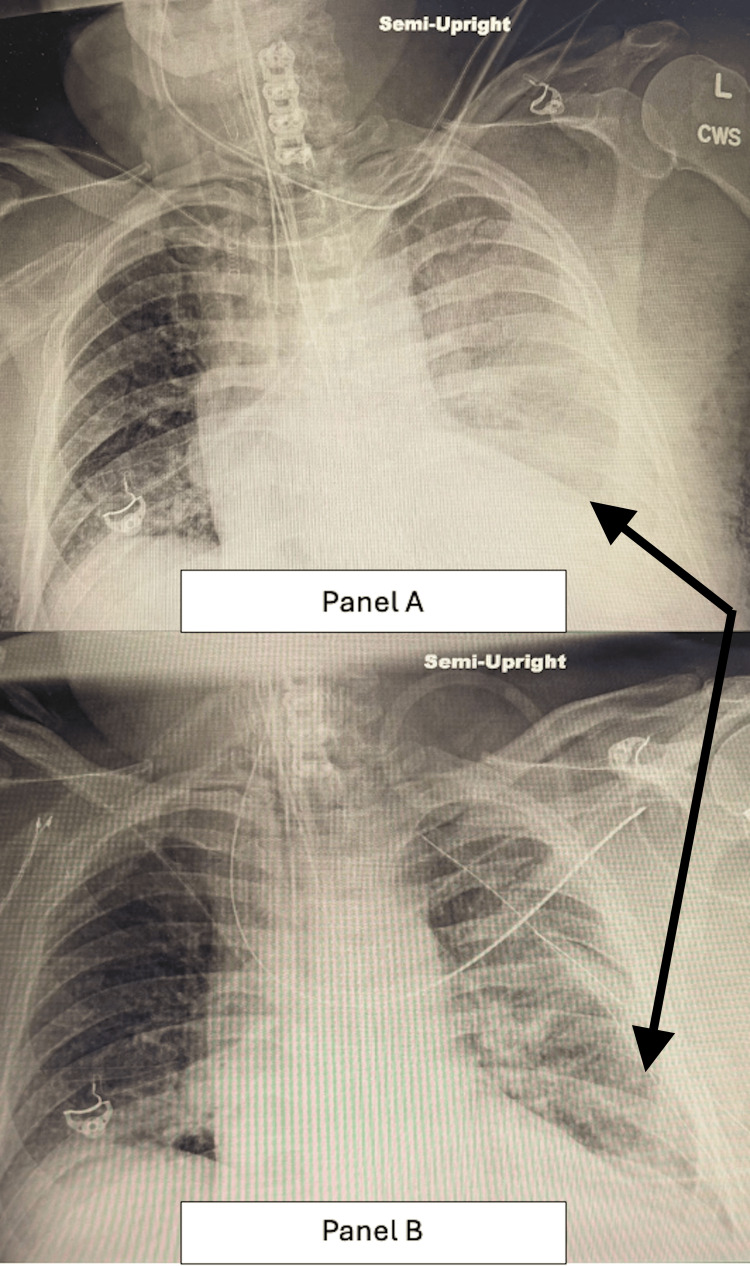
Chest X-rays before and after chest tube placement showing improvement with residual left pleural effusion. (A) Chest X-ray prior to chest tube placement with bilateral infiltrates (diffuse on the left and involving the lung base on the right) and left pleural fluid (black arrow). (B) Chest X-ray one day status post chest tube placement, improved from previous, with stable bilateral infiltrates and small residual left pleural effusion (black arrow). CWS: cervical spine view.

The left chest was prepped, and 5 cc of 1% lidocaine with epinephrine was infiltrated around the fourth intercostal space in the anterior axillary line. A 10-blade scalpel was used to make an incision, and a curved hemostat was advanced into the thoracic cavity, leading to the immediate expulsion of old, bloody fluid. A 28-French straight chest tube and a 26-French angled chest tube were placed and secured to the chest wall with 0-Prolene. Both tubes were connected to a closed chat drainage system and maintained at -20 cm H₂O suction, draining 1,000 cc of blood. A postoperative chest X-ray (Figures 6A, 6B) confirmed apical placement of the angled chest tube and mid-chest placement of the straight chest tube.

The chest tubes remained in place for eight days and were removed upon extubation. A repeat chest X-ray showed mild but improved bilateral infiltrates, with left lower lobe atelectasis or scarring, but no evidence of pleural fluid or pneumothorax. Pleural fluid cultures showed no bacterial growth. The patient continued to improve in the ICU and was later transferred to a step-down unit, where his condition further stabilized. After a total hospital stay of 23 days, he was discharged home in stable condition.

## Discussion

Case reports have examined the effectiveness of blowhole incisions with NPWT in treating SE, including cases of an unrestrained passenger in a motor vehicle accident (MVA), accidental strangulation, and opioid inhalation injury. A 60-year-old male was injured in a motor-vehicle accident while driving a van unrestrained, which resulted in fourth to ninth right-sided rib fractures and a bilateral pneumothorax. In addition to this, he had many other fractures in his thoracic vertebrae and a hip dislocation. Of note, the patient had undergone a left lower lobectomy due to cancer 12 years prior to the accident. Both thoracic cavities had tube placements for drainage, and the patient subsequently became dependent on mechanical ventilation. SE developed and extended to his face, neck, thoracic, and abdominal cavities, including into his pelvis. He received bilateral subclavicular blowhole incisions with negative pressure wound therapy (NPWT) [4]. In this case, a singular blowhole incision was initiated first; however, problems with continuous suction necessitated bilateral blowhole incisions. This potentially could have been initiated from the start, based on the presenting injuries affecting both lungs as well as his past medical history, resulting in an already reduced respiratory function. Thoracic surgical intervention for this patient would have been definitive treatment for the underlying cause of the SE; however, given his left lower lobectomy along with his other injuries that also needed surgical intervention, blowhole incisions were the safer option and still resulted in respiratory recovery.

Blowhole incisions with NPWT offer a simple yet effective solution in certain cases. A 45-year-old male presented with massive progressive SE from the periorbital area to the upper mediastinum, following a tracheal injury from blunt trauma, was initially managed conservatively due to the classification of his injuries as “minor laryngo-tracheal trauma” [7]. Despite unremarkable labs and imaging, his SE worsened by the second day, resulting in increased neck girth and dyspnea, classifying him as grade 5. Expectant management was initiated, and surgical management was avoided as it could cause more damage due to his injury, resulting in a fibrotic patch below the cricoid with no evidence of tear. The patient recovered within a month after blowhole incisions were performed [7]. In this case, drastic interventions were contraindicated as the injury was sustained to the neck, with drain placements being of no benefit. This case emphasizes the importance of blowhole incisions with NPWT, especially when more conservative interventions are required.

In addition to the benefits of this approach to resolve SE, blowhole incisions with NPWT can also treat the underlying cause of the SE. A 30-year-old man with a history of polysubstance abuse presented with dyspnea due to inhalation of fentanyl and heroin. On arrival, he had a respiratory rate of 36 per minute and an O_2_ saturation of 81% on room air and he was placed on a 15L non-rebreather facemask. His imaging showed extensive pneumomediastinum with no evidence of a pneumothorax. After this, his condition deteriorated requiring emergent intubation and vasopressor support. Cardiothoracic surgery was consulted and opted to perform a bedside right-sided blowhole incision with placement of a vacuum resolving the pneumomediastinum and SE within two days [8]. By draining the mediastinal air, this approach was the least invasive option and was the definitive treatment for his pneumomediastinum while also resolving the extensive SE. Utilizing blowhole incisions with NPWT can therefore treat the underlying cause while simultaneously treating the resultant SE minimizing the need for multiple, and possibly complex, procedures.

Blowhole incisions are typically performed in the infraclavicular area or another accessible location where air is trapped, and they allow for direct expulsion of the trapped air. In our case, the incision was made below the right clavicle, followed by dissection to create a pocket down to the pectoralis fascia, facilitating the release of air and reducing the tension on the surrounding tissues. The creation of a pocket in the subcutaneous tissue allows for a more controlled release of air, reducing the risk of damage to underlying structures while alleviating the respiratory distress associated with subcutaneous emphysema.

Studies have shown that early surgical intervention, such as blowhole incisions, can significantly improve outcomes and is linked to faster resolution of SE and improved respiratory function in patients with severe chest trauma, especially with extensive emphysema unresponsive to conservative management [9]. Additionally, blowhole incisions have proven effective in treating severe pneumomediastinum and SE, especially in cases with significant air accumulation [10].

Negative pressure wound therapy (NPWT) has been increasingly utilized in the management of subcutaneous emphysema and pneumomediastinum to accelerate air evacuation and reduce complications. NPWT applies controlled negative pressure to a wound or incision site, promoting drainage of fluids and air, thereby facilitating the healing process. In this case, NPWT was used following the blowhole incision to help evacuate residual air and fluids from the subcutaneous tissue, further aiding the patient's recovery.

A recent review demonstrated the efficacy of NPWT in treating complex wounds, including those associated with subcutaneous emphysema and pneumomediastinum. The use of NPWT in these cases allows for continuous removal of trapped air and reduces the risk of reaccumulation, thereby promoting faster resolution of the condition and improving respiratory function. The authors found that NPWT not only accelerates wound healing but also significantly reduces infection rates, which is particularly important in patients with traumatic injuries [11].

While blowhole incisions and NPWT are generally considered safe and effective, they come with certain risks. One potential complication is the risk of infection, particularly when the wound is left open for air expulsion. Proper wound care and monitoring are essential to minimize this risk. Additionally, blowhole incisions must be made carefully to avoid vital structures such as blood vessels and nerves. In this case, attention to anatomical landmarks during dissection helped ensure that no complications arose. Despite the advantages of this approach, it is important for clinicians to be aware of these potential risks and to act promptly if complications arise.

Furthermore, while this case demonstrated the effectiveness of blowhole incisions and NPWT, the timing and placement of such incisions require careful consideration. As discussed in the literature, early intervention tends to yield better outcomes, particularly in patients with progressive emphysema or pneumomediastinum. However, the optimal timing for surgical intervention and the role of NPWT in specific clinical settings remain subjects for further research.

## Conclusions

In conclusion, this case highlights the utility of blowhole incisions in the management of severe subcutaneous emphysema and pneumomediastinum following traumatic chest injury. The procedure, when combined with NPWT, provides an effective means of relieving respiratory distress, improving patient outcomes, and expediting recovery. The literature supports the use of blowhole incisions in patients with extensive air accumulation that has failed to respond to conservative management. Further studies are needed to refine the indications, timing, and optimal techniques for this intervention. The successful application of this technique in this case emphasizes the importance of early surgical intervention and multidisciplinary management in patients with severe chest trauma.
